# Transcranial direct current stimulation to modulate brain reactivity to food cues in overweight and obese adults: study protocol for a randomized controlled trial with fMRI (NeuroStim-Obesity)

**DOI:** 10.1186/s13063-022-06234-8

**Published:** 2022-04-12

**Authors:** Peyman Ghobadi-Azbari, Nastaran Malmir, Meghedi Vartanian, Rasoul Mahdavifar-Khayati, Somaye Robatmili, Venus Hadian, Sara Derafsheh, Michael A. Nitsche, Masoud Nosratabadi, Ali Farhoudian, Hamed Ekhtiari

**Affiliations:** 1 Department of Biomedical Engineering, Shahed University, Tehran, Iran; 2 Iranian National Center for Addiction Studies (INCAS), Tehran University of Medical Sciences, Tehran, Iran; 3 Department of Psychology, Islamic Azad University, Science and Research Branch, Tehran, Iran; 4 Department of Clinical Psychology, Tehran University, Tehran, Iran; 5 Department of Cognitive Neuroscience, Tabriz University, Tabriz, Iran; 6 Department of Psychology and Neurosciences, Leibniz Research Centre for Working Environment and Human Factors, Dortmund, Germany; 7 Department of Neurology, University Medical Hospital Bergmannsheil, Bochum, Germany; 8 Department of Psychology, Paarand Center for Human Enhancement, Tehran, Iran; 9 Department of Psychiatry, Tehran University of Medical Sciences, Tehran, Iran

**Keywords:** Obesity, Food craving, Transcranial direct current stimulation (tDCS), Functional magnetic resonance imaging (fMRI), Diffusion tensor imaging (DTI), Dorsolateral prefrontal cortex (DLPFC)

## Abstract

**Background:**

With increasing obese populations worldwide, developing interventions to modulate food-related brain processes and functions is particularly important. Evidence suggests that transcranial direct current stimulation (tDCS) over the dorsolateral prefrontal cortex (DLPFC) may modulate the reward–control balance towards facilitation of cognitive control and possible suppression of reward-related mechanisms that drive food cue-induced craving. This protocol describes a clinical trial that investigates the neurocognitive mechanisms of action for tDCS to modulate food cue-reactivity and cravings in people with obesity.

**Method:**

The NeuroStim-Obesity trial is a prospective, randomized, sham-controlled, double-blind single-session tDCS trial targeting food craving in those with obesity or overweighed. Once randomized, 64 adults with obesity or overweighed complete one session in which they receive either active or sham tDCS over the DLPFC (anode F4 and cathode F3, 2 mA intensity for 20 min). The primary outcome is change in neural response to the food cue-reactivity task in the ventral striatum after a single-session bilateral tDCS compared to sham stimulation. Secondary outcomes include changes in food craving evaluated by the Food Craving Questionnaire-State (FCQ-S). We will also explore the predictive role of brain structure and functional networks assessed by structural and functional magnetic resonance imaging (MRI) during both task performance and the resting-state that are acquired pre- and post-intervention to predict response to tDCS.

**Discussion:**

The results will provide novel insight into neuroscience for the efficacy of tDCS and will advance the field towards precision medicine for obesity. Exploratory results will examine the potential predictive biomarkers for tDCS response and eventually provide personalized intervention for the treatment of obesity.

**Trial registration:**

Iranian Registry of Clinical Trials (IRCT) IRCT20121020011172N4. Retrospectively registered on 4 June 2020

## Background

Given the worldwide increase of the proportion of overweight or obese adults, the development of interventions to modulate food-related brain processes and functions is of great scientific and public interest [1]. There is a large body of existing evidence that studies the correlation of BMI with neural, behavioral, or psychological parameters [2–7]. BMI has been also positively associated with food craving [8–10]. Food craving plays an important role in the etiology of overweight and obesity with increasing total energy intake [11, 12]. Such craving is a form of food cue-reactivity: a conditioned appetitive response to food cue that is mostly accompanied by increased salivation [13] and neural activity in gustatory and reward-relevant brain areas [14]. As such, food craving and other forms of food cue-reactivity may function as conditioned responses that serve as triggers for increased food consumption [15] and weight gain [16], which may exacerbate the risk for rising obesity rates [17]. Albeit there are positive outcomes in controlling obesity using nutritional, psychological, and pharmacological approaches, a majority of patients with frequent food cravings manifest a chronic, relapsing course of disease within 1 year of obesity treatments [18–20] resulting in a need for alternative or adjuvant treatment options. Therefore, non-invasive neuromodulation approaches have been extensively investigated to regulate food craving by modulating the relevant dysregulated networks and neural activity as a possible approach to organize the decision-making process and control the behaviors of food cravers [21–24]. In this context, transcranial direct current stimulation (tDCS) has aroused particular interest in research and practice in recent years.

In order to determine the neurophysiological, cognitive, or clinical effects of non-invasive brain stimulation (NIBS), most studies compare the effects of active stimulation with a sham intervention. Hence, sham tDCS protocols are fundamental because of the placebo response observed in NIBS trials [25] and the fact that nonblinded trials overestimate the effects of subjective and objective results [26].

The therapeutic potential of tDCS intervention for obesity is hypothesized to be based on the modulation of dysregulated top–down control by prefrontal cortex (PFC) function and reward processing in subcortical-limbic structures, mainly the ventral striatum. In recent years, data derived from neuroimaging and cognitive assessments provide evidence that food cue-reactivity, particularly in obese persons, is associated with dysregulated activities within the prefrontal regions, specifically, the dorsolateral prefrontal cortex (DLPFC). The potential for DLPFC neuromodulation might remediate these dysregulated activities within prefrontal brain regions that have been associated with both impaired inhibitory control (i.e., binge eating and purging) and poor cognitive flexibility (e.g., the obsessive concerns with eating, weight, and shape) [12, 22–24].

Accordingly, therapeutic strategies that modulate brain activity in the DLPFC might regulate or suppress food cue-reactivity, cravings, and appetite. Examining the effect of cortical activity modulation using high-frequency rTMS over the left DLPFC in eating disorders and obesity was tested in many studies and a temporary anti-craving effect of this modulation has been demonstrated [27, 28]. Another clinical study also reported beneficial effects of using high-frequency rTMS applied over the left DLPFC to modify cue-induced food cravings and binge eating in people with a bulimic eating disorder [29]. This finding was replicated using bilateral tDCS to the DLPFC (anode over F4/cathode over F3), which demonstrate a reduction in self-reported cravings for active stimulation as compared to sham stimulation [30–32].

However, no study has evaluated the neurofunctional mechanisms of action of tDCS on food cue-reactivity. Therefore, the present study was designed as a randomized parallel-design, sham-controlled, double-blind trial to investigate the neurofunctional mechanisms of action for tDCS to modulate food cue-reactivity and cravings in ones with obesity or overweight problems using fMRI. We focused on the following aims in this trial:

1. To determine whether tDCS over DLPFC changes subcortical-limbic reactivity to food cues in obesity
2. Whether tDCS could be an effective intervention for reducing food craving
3. To determine how tDCS affects neural correspondence of craving using fMRI
4. To determine the efficacy of tDCS on suppression of craving that influenced by individual baseline differences
5. To explore which of (a) subjective, (b) clinical, (c) behavioral, (d) structural, or (e) neural circuits measures predict tDCS induced craving reduction in obesity

## Methods/design

### Design and setting

This study is conducted according to a double-blind randomized sham-controlled design, and it is a single-center superiority trial, which was conducted at the National Brain Mapping Lab, Tehran, Iran. The study is currently recruiting and 64 subjects with obesity or overweighed between the age of 18 and 61 years are to be randomly divided into two parallel arms which deliver active or sham DLPFC tDCS. A single-session bilateral tDCS over the DLPFC is applied in half of the subjects while the other half receives sham stimulation.

**Fig. 1:**
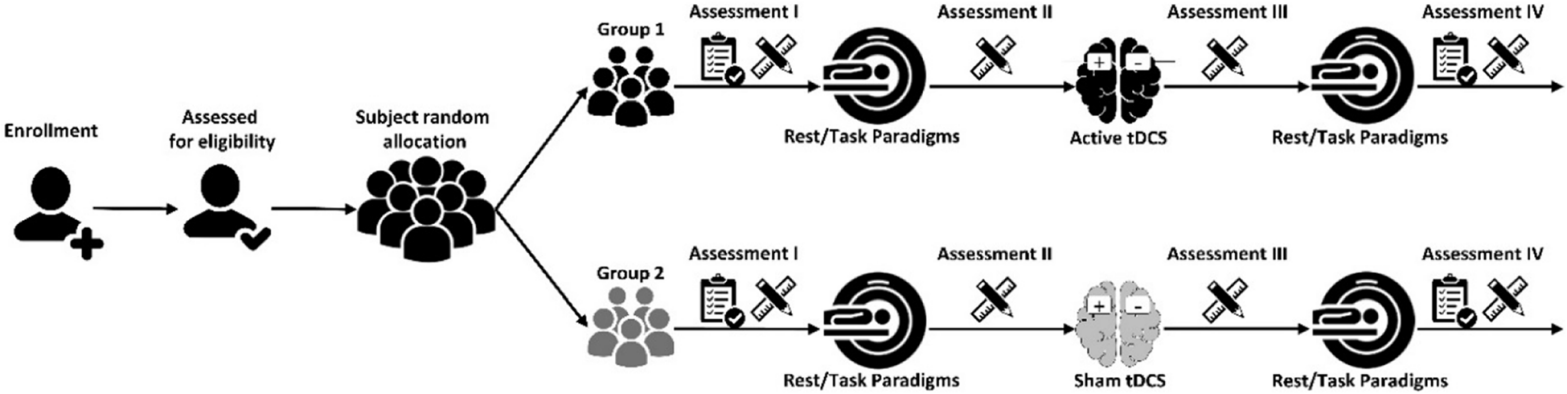
The NeuroStim-Obesity flowchart

The course of the trial is visualized in Fig. 1.

After signing the consent form, eligible participants undergo intensive baseline assessments using self-report and clinical assessments. Participants in the active arm receive 2 mA anodal tDCS for 20 min over the right DLPFC with the cathode over the left DLPFC. The set-up for sham and real stimulation sessions is exactly the same. Structural, resting-state (rs), and task-based functional MRI (pictorial food cue exposure) was done immediately before and after active or sham tDCS. Immediate craving is assessed with a single item 0–100 VAS question at multiple time points before and after imaging and tDCS. The Ethics Committee of Iran University of Medical Sciences approved the research to trial (IR.IUMS.REC.1396.0459), which is registered with the Iranian Registry of Clinical Trials (IRCT20121020011172N4).

### Plans to promote participant retention

In order to promote retention, we have prepared a video about how to execute the project and details of the project process to reduce possible anxiety and answer the questions. Participants can watch it before testing day, and they are encouraged to share their questions or concerns. All participants are welcome by one of the trained researchers in the testing day and they were all accompanied until the end of the experiment. Any adverse effect during the study is recorded in detail, and they also have the contact number of researchers for advice in case of any possible side effect in the following days. They are informed that they have the option to withdraw from the study at any time. If this occurs, their data will be removed from the result of the study.

### Participants

We plan to enroll 64 subjects from specialized nutrition clinics by flyer advertising. We also made a video to get people more familiar with our project. Participants must follow all of the inclusion criteria below:

1. Age ≥ 18 and < 61 years old
2. Right-handedness
3. Persian speaking
4. BMI: 25–35 kg/m^2^
5. Frequent food cravings (≥ 3 per day during last month, as assessed by self-report questionnaire)
6. Being responsive in food cue-reactivity screening at baseline (defined as mean craving scores (> 70) in food-related images on the Visual Analog Scale (0–100))

The participant with any of the following criteria will be excluded from the study:

1. Unwillingness or inability to complete any of the major aspects of the study protocol including food cue rating or behavioral assessments
2. Comorbid psychiatric disorders (i.e., depressive, bipolar, or psychotic disorders), which are evaluated by a mental health specialist
3. Active suicidal ideation with intent or plan as determined by self-report or assessment by a mental health specialist during the initial screening or any other phase of the study
4. Unstable medical disorder reported in subject’s medical history or by a clinician assessment
5. Non-correctable vision or hearing problems
6. Personal or family history of seizures
7. History of stroke
8. Any other condition the research team feel would put the subject at risk for entering the study
9. Contraindication to tDCS (pacemaker, a metal embedded in the scalp or brain, skin lesions at the site of stimulation, and history of head injury or neurosurgery) and MRI (claustrophobia, metallic implants, ferromagnetic metals in the scalp or brain, and pregnant women)

Concomitant care and interventions prohibited during the trial will be the recent use of diabetes medication or insulin injection or a medical indication for use during the study period, the use of medications that can affect appetite or weight (e.g., orlistat, sibutramine, topiramate, bupropion), and participation in weight loss program or non-invasive brain stimulation therapy during the study.

If all eligibility criteria are reached and participants provide written informed consent for study participation, they will be included in the study sample. Eligibility will be determined by the trained study staff during screening and baseline assessments.

### Intervention

Transcranial direct current stimulation will be administered via a battery-driven stimulator (DC-Stimulator Plus, Neuroconn GmbH, Germany). Direct current will be transferred by two saline-soaked sponge electrodes (7 × 5 cm^2^). For effective bilateral tDCS, the tDCS montage will comprise placement of the anode over the right DLPFC and the cathode over the left DLPFC which corresponds to the F4 and F3 areas, according to the 10–20 EEG system. Active tDCS will be delivered with a constant current of 2 mA for 20 min (ramp-up/down: 30 s). The montage and dose of stimulation were chosen based on previous studies for food craving in healthy adults [30–32]. For the sham group, the same electrode position and ramp-up/down time will be used, but the 2-mA current will be delivered only during the first 1 min of the 20-min stimulation period to elicit a transient tingling sensation on the scalp and to blind subjects as to the respective stimulation condition. Participants will be told about a mild tingling and itching sensation under the electrodes during the stimulation. After each active or sham tDCS session, participants will complete an adverse-effect questionnaire [33].

### Outcome measures and assessments

**Fig. 2:**
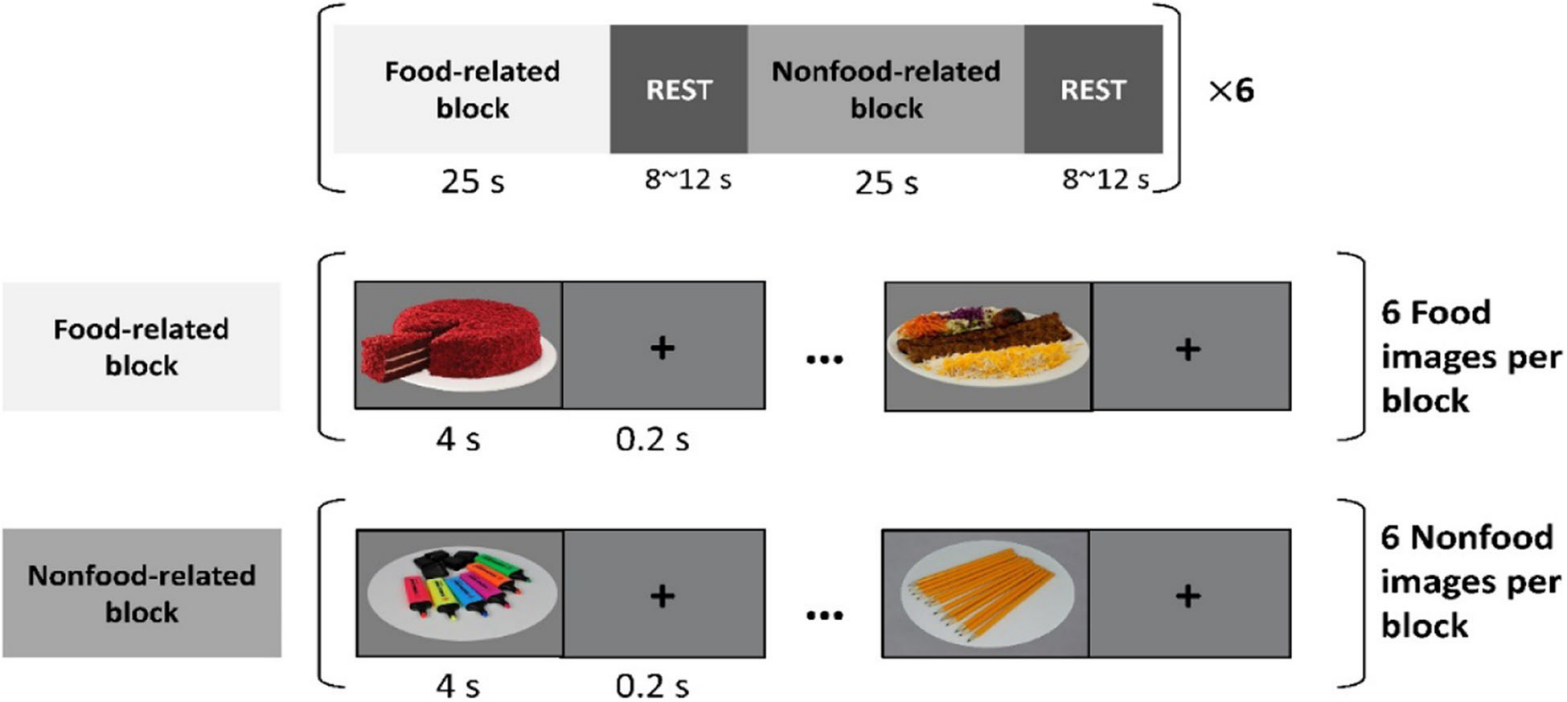
Structure of the food cue-reactivity task. The task included food blocks and non-food blocks

Outcome measures for the food cue-reactivity task (see Fig. 2) will be acquired pre- and post-fMRI at each session. At pre- and post-assessments, additional outcome measures targeting neural substrates will be assessed. The timeline of assessment measures is shown in Table 1. Each outcome measure will be analyzed regarding potential comparisons between the study groups (active vs. sham tDCS).

**Table 1:** Schedule of enrollment, interventions, and assessments

			Post-allocation							
			Baseline	Pre_**fMRI1**_	fMRI_**1**_	Post_**fMRI1**_	Intervention	Pre_**fMRI2**_	fMRI_**2**_	Post_**fMRI2**_
			~1 h	~10 min	~30 min	~10min	30 min	~10 min	~30 min	~25 min
Time point	Measurement	Mode	T0	T1	T2	T3	T4	T5	T6	T7
Enrollment										
Eligibility screening		Paper	X							
Informed consent		Paper	X							
Neuropsychological screening	Demographic data	Paper	X							
	EHI [34]	Paper	X							
	CES [35]	Paper	X							
	DASS-21 [36]	Paper	X							
	TFEQ-R-18 [37]	Paper	X							
	EDDS [38]	Paper	X							
	Food craving pictures	Tablet-PC	X							
Intervention							**↔**			
Viewing task	Food cue-reactivity	Computer			X				X	
Brain stimulation	tDCS (active vs. sham)	Device					X			
Questionnaires	FCQ-T [39]	Paper		X						X
	FCQ-S [40]	Paper		X						X
	Self-reported craving (VAS)	Paper		X		X		X		X
	Baseline hunger (VAS)	Paper		X		X		X		X
	Seven affective states (VAS)	Paper		X		X		X		X
	Blindness	Paper								X
	Sensations related to tES [33]	Paper								X
Additional assessments										
Physical measures	fMRI	Computer			X				X	
	DTI	Computer								X

*Abbreviations: EHI* Edinburgh Handedness Inventory, *CES* Compulsive Eating Scale, *DASS-21* Depression Anxiety Stress Scales-21, *TFEQ-R18* Three-Factor Eating Questionnaire-R18, *EDDS* Eating Disorder Diagnostic Scale, *FCQ-T* Food Craving Questionnaire-Trait, *FCQ-S* Food Craving Questionnaire-State, *VAS* Visual Analog Scale, *DTI* Diffusion tensor imaging

#### Primary outcomes

The primary outcome is the changes from pre-fMRI to post-fMRI in VAS scores (for food craving) at post-tDCS compared to changes at pre-tDCS in the sham and active tDCS groups.

#### Secondary outcomes

- Change in FCQ-S scores from before tDCS to after tDCS, as measured by Food Craving Questionnaire-State [40]
- Change in resting-state functional connectivity between cortical and subcortical regions, as assessed by correlation between spontaneous BOLD signal fluctuations in subcortical ROIs and voxels within the prefrontal cortex and insula from before tDCS to after tDCS
- Change in resting-state functional connectivity under the stimulated area, as assessed by correlation between spontaneous BOLD signal fluctuations in the cortical area under the anode/cathode electrodes and whole brain from before tDCS to after tDCS
- Change in task-based functional activation in the prefrontal cortical areas and subcortical-limbic areas, as assessed by the BOLD signal changes with voxel-wise analysis in the regions of interests (ROIs) (prefrontal cortex, insula, thalamus, ventral striatum, and extended amygdala) from before tDCS to after tDCS
- Change in task-based functional connectivity between the cortical and subcortical regions, as assessed by psychophysiological interaction (PPI) between spontaneous BOLD signal fluctuations in subcortical ROIs and voxels within the prefrontal cortex and insula from before tDCS to after tDCS
- Change in task-based functional connectivity under the stimulated area, as assessed by psychophysiological interaction (PPI) between spontaneous BOLD signal fluctuations in the cortical area under the anode/cathode electrodes and whole brain from before tDCS to after tDCS
- Change in Resource Allocation Index (RAI) in rs-fMRI, as assessed by correlation among default mode network (DMN), saliency network (SN), and executive control network (ECN) in rs-fMRI from before tDCS to after tDCS [41]

#### Exploratory outcomes

Exploratory analyses will be conducted for measures of subjective, behavioral, neuropsychological, or neural circuits which will be analyzed for identifying potential predictors of food cue-reactivity task performance, and responsiveness to the intervention, as measures by an exploratory regularized regression model. Additionally, the induced E-field in the prefrontal area, derived from the analysis of the computational finite-element model, will be included as a potential predictor of neural response to tDCS [42, 43].

### Outcome assessments

The study timeline shown in Table 1 provides an overview of the time schedule of enrollment, interventions, and assessments.

#### Baseline assessments

At baseline assessment (T0), participants will be given written informed consent and complete a demographic questionnaire, a handedness inventory [34], Depression Anxiety Stress Scales-21 (DASS-21 [36]), Eating Disorder Diagnostic Scale (EDDS [38]), Compulsive Eating Scale (CES [35]), and Three-Factor Eating Questionnaire-R18 (TFEQ-R18 [37]). Afterward, participants will perform the food cue-reactivity training task to learn about the actual fMRI task (see the “Magnetic resonance imaging” section), which consists of one practice trial with 6 images of food and non-food products. The baseline assessment will take approximately 1 h.

#### Pre- and post-assessments

The 0–100 continuous VAS will provide the possibility for assessing the self-reported food craving, hunger, and affective states in multiple time points before and after fMRI and tDCS (T1, T3, T5, and T7). Furthermore, FCQ-S and FCQ-T will provide the possibility for assessing the intensity of trait and state dimensions of food cravings before the first fMRI (T1: pre-tDCS) and after the second fMRI (T7: post-tDCS). Finally, participants will complete a questionnaire to assess blindness and potential adverse events of tDCS (T7).

### Magnetic resonance imaging

MRI will be assessed at the Iranian National Brain Mapping Laboratory with a 3 Tesla scanner (Siemens Prisma) using a 20-channel head coil, prior to and immediately after intervention (see Table 2 for MRI data acquisition parameters).

**Table 2:** MRI data acquisition parameters

Sequence	Main parameters
Resting-state fMRI	*TR = 2500 ms, TE = 30 ms, FOV = 192 × 192 mm*^*2*^*, 43 slices, 144 volumes, 3.0 × 3.0 × 3.0 mm*^*3*^*, flip angle = 90°*
Task-based fMRI	*TR = 2500 ms, TE = 23 ms, FOV = 192 × 192 mm*^*2*^*, 43 slices, 167 volumes, 3.0 × 3.0 × 3.0 mm*^*3*^*, flip angle = 70°*
DTI	*TR = 8000 ms, TE = 68 ms, 70 slices, 2 × 2 × 2 mm*^*3*^*, 64 directions (b = 1000 s/mm*^*2*^*)*
T1w	*TR = 1810 ms, TE = 3.45 ms, TI = 1100 ms, 176 slices, 1.03 × 1.03 × 1.0 mm*^*3*^*, flip angle = 7°*
T2w	*TR = 3200 ms, TE = 408 ms, 176 slices, 0.45 × 0.45 × 0.9 mm*^*3*^*, flip angle = 120°*

*Abbreviations: TR* Repetition time, *TE* Echo time, *TI* Inversion time, *FOV* Field of view, *fMRI* Functional magnetic resonance imaging, *DTI* Diffusion tensor imaging

**Fig. 3:**
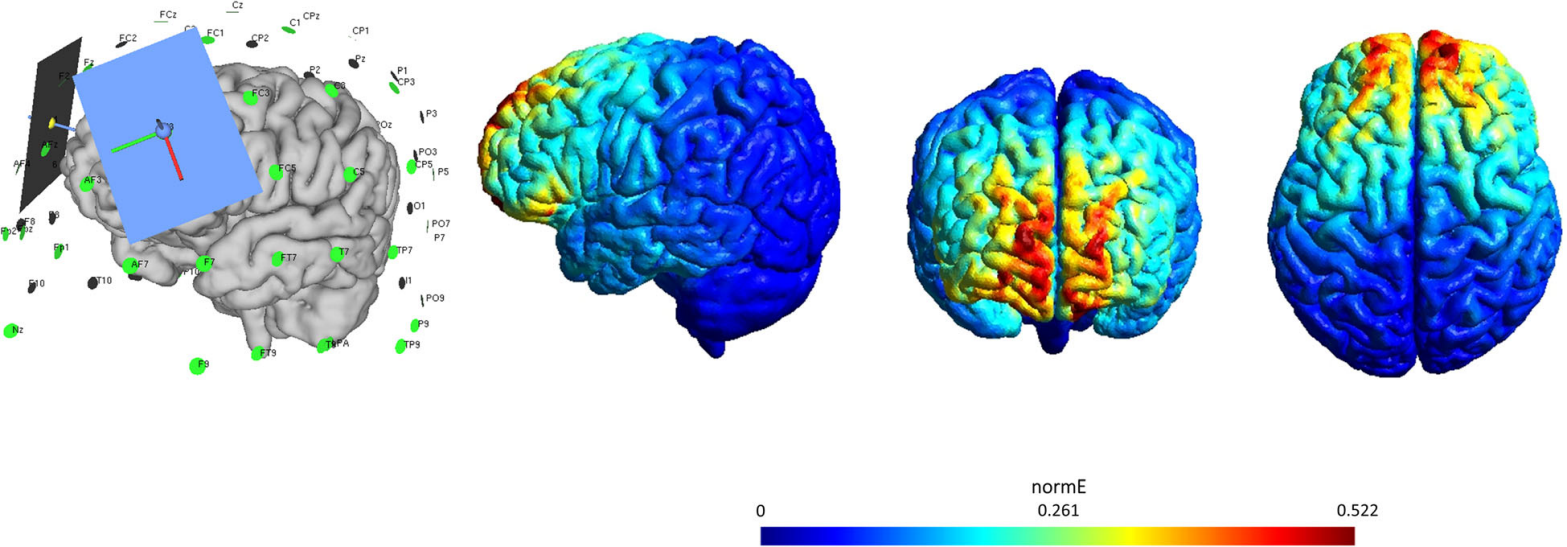
Electrode positioning (anode, F4; cathode, F3) and computational model of the E-field induced in tDCS using SimNIBS, for one example participant (f, 27 years)

Both MRI sessions include structural T1-weighted acquisition as well as resting-state fMRI to assess functional connectivity within and between large-scale networks that mediate food craving. Furthermore, a food cue-reactivity fMRI task will be used to assess intervention-associated changes in prefrontal functioning (see Fig. 2). At the end of the post-tDCS MRI assessment, additional T1- and T2-weighted images will be acquired with parameters optimized for computational finite-element method (FEM) modeling to calculate electric field strength and distribution induced by tDCS [44, 45]; see Fig. 3 for sample modeling analysis. After the MRI sessions, a separate DTI scan will be also performed to assess the structural integrity of the cortical-subcortical tracts, via quantification of fractional anisotropy [46]. In the food cue-reactivity task, participants will view 12 blocks of 6 images each (6 blocks with foods and 6 blocks with non-foods). Each block consists of 6 images of either food or neutral valence presented for 4000 ms with an interstimulus interval of 200 ms followed by an inter-block interval (i.e., gray screen with crosshair) with a duration (8–12 s). They will be given the following task instruction: “In the next task you will see food and non-food products. Please look at the images and pay close attention, since during the MRI session your job will be to press a button on the scroll wheel whenever you see the yellow border images.”

Prior to beginning enrollment for this protocol, a pilot study to determine food cue validity will be completed. Twenty people with obesity or overweight problems will participate in a validation phase to evaluate a database of food-related pictures. Participants will be invited from specialized nutrition clinics with flyer advertising. After signing the consent form, participants will be screened for psychiatric conditions and eating disorder history. Participants will be invited for a 90-min session where they will be asked to see a randomized series of 120 food-related pictures and 120 neutral pictures on the computer screen and answer the following questions presented consecutively under each image in 4 domains.

(1) Valence: After seeing this picture, please describe your mood, from negative to positive on a 1 to 9 self-assessment manikin scale.

(2) Arousal: After seeing this picture, please describe the level of arousal, from calm to excited on a 1 to 9 self-assessment manikin scale.

(3) Craving: How much can this picture induce food craving in an overweight or obese adult (from 0 to 100 on a Visual Analog Scale).

(4) Typicality: How frequently does an overweight or obese adult see scenes like this image during his/her food use (from 0 to 100 on a Visual Analog Scale).

Two sets of 36 pictures from this database, one set from food and one set from neutral pictures, will be randomly selected with a computer R-script to have equal values for brightness and hue, craving association, and frequency. These sets will be used for the food cue-reactivity fMRI task inside the scanner.

### Safety outcome

Subjects will be fully informed about the foreseeable risks and discomforts associated with participation in this study. The consent forms describe these risks and discomforts clearly. Patients must know that they have the option to withdraw from the study at any time. Withdrawal from this study can be done without consequence. The investigators may also choose to terminate a participant from this study, if they suffer a severe adverse event, do not follow study requirements, or feel that continued participation would put the person at a greater risk than indicated. Headaches, itching, and paresthesia are generally very mild with tDCS and are limited to the actual treatment duration. Since skin nerves habituate to the electrical stimulation rather quickly, most subjects are not aware of the stimulation after about the first 1 min. This is what allows sham stimulation to be effectively masked. More persistent headache can be treated with acetaminophen or ibuprofen. The range of stimulation intensities for human studies of tDCS is usually 1–2 mA as we do in our study. Skin redness is common with tDCS studies because the electrical stimulation increases local blood flow under the electrodes. This redness should dissipate within 30 min or less. There should be no evidence of redness or skin breakdown before the tDCS application.

### Sample size estimation

Based on a recent study in the field using single-session bilateral tDCS over the DLPFC compared to sham tDCS [31], we estimated an effect size of 0.55. To demonstrate the intervention effect in the primary outcome, 64 participants (32 per tDCS group) need to be included in the analysis with a two-group *t*-test with a 0.05 two-sided significance level and a power of 80%. Sample size estimation was conducted using PASS software [47].

### Blinding and randomization

During the recruited process, participants are informed that the study evaluates neural responses to the transcranial direct current stimulation but are not told anything about sham tDCS and differences between them. The screen of the tDCS device is hidden from participants and the group allocation remains indistinguishable to both participants and the investigators. The sham tDCS condition consists of a 30-s ramp-up to 2 mA and immediately ramp-down to 0.0 mA over 30 s to let participants feel the itching resembling to the real stimulation. To ensure the success of blinding, we will ask participants at the end of the study to guess whether the stimulation was real or sham.

We use the sealed envelope method (see https://www.sealedenvelope.com) to group assignment (i.e., either active tDCS or sham tDCS) at the beginning of the study (by NM). All participants will have a unique ID during the study process, and only an independent clinician who is not involved in the study knows them (NS). Main investigators and participants are kept blind to the allocation of study groups.

A blocked randomization (6-block) will be used to balance the number of participants of the two groups that are run in parallel. Each subject randomly assigns to each of active or control DLPFC tDCS. However, blinding could be unlocked if requested by the participant or trained researchers due to any possible side effect. If this happens, the data is considered unconfirmed data and will not entry to analysis.

### Quality control and data management

The research team is comprised of the principal investigator (HE); two professionally trained researchers (NM and PGA), who composed the steering committee; and research assistances (MV, VH, SD) that have regular weekly meetings to ensure about consistency on project administration details (e.g., subject recruitment, data curation, etc.) and discuss about important issues and trial progress. The data collected in this trial will comprise neuroimaging data and psychological questionnaires. Two professionally trained researchers (NM and PGA) are responsible for collecting confirmed data and data entry (under the supervision of HE).

### Statistical methods

Baseline participant characteristics will be presented as mean ± standard deviation for continuous variables or percentages for categorical variables. Participant demographics data will be compared between the active tDCS and sham tDCS groups, using a two-sample *t*-test (Student’s *t*-test).

To control for the tDCS intervention efficacy, a repeated-measures analysis of variance (ANOVA) will be performed on FCQ-T with time (before/after tDCS) as the within-subject factor and group (active tDCS/sham tDCS) as the between-group factor. The same strategy will be followed for behavioral and neuropsychological data.

Changes in functional neural parameters (resting-state and task-related fMRI) will be preprocessed and analyzed using the Analysis of Functional NeuroImages (AFNI) [48]. To assess brain–behavior associations, the correlations between changes in brain activity/connectivity and subjective craving will be investigated using the Pearson correlation test. As an exploratory analysis, the definition of major prognostic factors for response to active tDCS will be assessed using the exploratory regularized regression model. Assessment of the model validity will be performed using leave-one-out cross-validation within this dataset and will be validated with subsequent test datasets in the future studies. All statistical analyses will be run using the R Statistical Package [49]. There will be no interim analysis. The investigators will be empowered to stop the trial under the following circumstances:

- Harm
  - There is a significant increased risk for serious adverse events, including severe itching and redness at the electrode site, nausea, dizziness, or drowsiness.
- Safety
  - Significant safety concerns emerge and the Research Ethics Committee of Iran University of Medical Sciences choose to pause or stop the trial.

We do not plan to conduct additional analyses, both subgroup and adjusted analyses.

We expect very low missing data in our baseline neuropsychological covariates and in the food craving measures (Table 1). Missing data will be reported in the publication. More than 5% missing data will result in multiple imputation with the generation of 40–50 imputed data sets to be analyzed separately and then aggregated into one estimate of the effect of the intervention on the primary and secondary outcomes. Missing data will be supplemented by the Multivariate Imputation by Chained Equations (MICE) method [50, 51].

## Discussion

This randomized controlled trial with two parallel groups will investigate the effects of a single session of bilateral tDCS on food cue-induced cravings as well as functional neural parameters in obese people with frequent food cravings. In the active group, bilateral tDCS (1 mA, 20 min) will be applied over the DLPFC (anode over the right DLPFC, cathode over the left DLPFC) while the control group will receive sham tDCS (1 mA, 30 s). Findings of this study will contribute to the understanding of predictive biomarkers, as well as the underlying mechanisms of tDCS effects in the brain response to food-related cues.

As discussed previously, prior studies indicated that prefrontal cortex tDCS modulated food cravings [30–32], and provided evidence that food craving is associated with DLPFC activity. The mechanistic neural substrates by which DLPFC stimulation decreases food cravings are unknown, although data suggest that the amount of activity in the right prefrontal cortex may determine the degree of inhibition over downstream circuits that promote overeating [52]. On the one hand, this trial aims to demonstrate the mechanistic neural substrates of tDCS in people with obesity. Resting-state and task-based fMRI will presumably allow us to examine mechanistic hypotheses for the modulatory role for prefrontal tDCS in both small-scale top-down regulation and large-scale network interactions. On the other hand, this study should make a contribution to the identification of predictive biomarkers of response to tDCS. Further explorations using machine learning methods concerning the personalized interventions will help to identify prognostic biomarkers of tDCS response aiming to improve tDCS methods in the individual level and to understand the mechanisms of action of tDCS on a predictive basis.

In conclusion, the current clinical trial will investigate how brain functional neural parameters at the network level may influence tDCS impact on food cue-reactivity and craving, and also address how the tDCS can modulate brain function with the hope of improving treatment outcome. Our study will provide new insights for the neuromodulatory treatments for obesity, by identifying functional activity/connectivity biomarkers of the clinical response to tDCS stimulation and hopefully contribute significantly to refine this method to allow the customization of therapeutic protocols in the individual level.

### Patient and public involvement

We will complete a pilot study to determine food cue validity. A group of 20 overweight or obese people will be asked to rate images for craving, valence, arousal, and typicality. This provides a resource of validated images for the food cue-reactivity task inside the scanner (details in the “Magnetic resonance imaging” section).

Patients will be involved in the study design, especially where side effects are an issue, as they can give a patient’s perspective on the balance between risks and benefits.

An original paper will be prepared to present the trial results at the proper time after the end of the study. Results of the study will be disseminated to all study participants through E-mail recorded at the time of enrollment. At the end of the study, we will inform the patients of the results of their brain map through patient private meetings and will offer patient referral to a weight management program.

### Trial status

This manuscript reflects protocol version 8.1, dated 7 May 2021. Recruitment of participants was due to commence in December 2019. However, due to the COVID-19 pandemic, this commenced in February 2021, and the trial is currently recruiting in Iran. It is expected that recruitment will be completed by August 2022.

## Data Availability

The steering committee (see quality control and data management) have full access to the dataset, and the analyzed data will be reported in peer-reviewed journals. Original data will not be shared publicly and maintained entirely confidential. The full protocol (e.g., food cue-reactivity task characteristics or statistical codes) will be available at the reasonable request of researchers from the corresponding author.

## Abbreviations

AFNI: Analysis of functional neuroimages
BMI: Body mass index
CES: Compulsive eating scale
CSTC: Cognitive science and technologies
DASS-21: Depression Anxiety Stress Scales-21
DMN: Default mode network
DTI: Diffusion tensor imaging
ECN: Executive control network
EHI: Edinburgh Handedness Inventory
EDDS: Eating Disorder Diagnostic Scale
FCQ-S: Food Craving Questionnaire-State
FCQ-T: Food Craving Questionnaire-Trait
FEM: Finite-element method
fMRI: Functional magnetic resonance imaging
FOV: Field of view
IRCT: Iranian Registry of Clinical Trials
MRI: Magnetic resonance imaging
PFC: Prefrontal cortex
PPI: Psychophysiological interaction
RAI: Resource Allocation Index
SN: Saliency network
tDCS: Transcranial direct current stimulation
TE: Echo time
TFEQ-R18: Three-Factor Eating Questionnaire-R18
TI: Inversion time
TR: Repetition time
VAS: Visual Analog Scale
